# Utility of the new Movement Disorder Society clinical diagnostic criteria for Parkinson's disease applied retrospectively in a large cohort study of recent onset cases

**DOI:** 10.1016/j.parkreldis.2017.04.006

**Published:** 2017-07

**Authors:** Naveed Malek, Michael A. Lawton, Katherine A. Grosset, Nin Bajaj, Roger A. Barker, Yoav Ben-Shlomo, David J. Burn, Tom Foltynie, John Hardy, Huw R. Morris, Nigel M. Williams, Nicholas Wood, Donald G. Grosset, PRoBaND clinical consortium

**Affiliations:** a Department of Neurology, Ipswich Hospital NHS Trust, Ipswich, United Kingdom; b School of Social and Community Medicine, University of Bristol, Bristol, United Kingdom; c Institute of Neurological Sciences, Queen Elizabeth University Hospital, Glasgow, United Kingdom; d Department of Neurology, Queen's Medical Centre, Nottingham, United Kingdom; e Department of Clinical Neurosciences, John van Geest Centre for Brain Repair, Cambridge, United Kingdom; f Institute of Neuroscience, University of Newcastle, Newcastle upon Tyne, United Kingdom; g Sobell Department of Motor Neuroscience, UCL Institute of Neurology, London, United Kingdom; h Reta Lila Weston Laboratories, Department of Molecular Neuroscience, UCL Institute of Neurology, London, United Kingdom; i Department of Clinical Neuroscience, UCL Institute of Neurology, London, United Kingdom; j Institute of Psychological Medicine and Clinical Neurosciences, MRC Centre for Neuropsychiatric Genetics and Genomics, Cardiff University, Cardiff, United Kingdom; k Department of Molecular Neuroscience, UCL Institute of Neurology, London, United Kingdom

**Keywords:** Parkinson's disease, Diagnosis, Phenotype, Criteria

## Abstract

**Objective:**

To examine the utility of the new Movement Disorder Society (MDS) diagnostic criteria in a large cohort of Parkinson's disease (PD) patients.

**Methods:**

Recently diagnosed (<3.5 years) PD cases fulfilling United Kingdom (UK) brain bank criteria in *Tracking Parkinson's*, a UK multicenter prospective natural history study were assessed by retrospective application of the MDS criteria.

**Results:**

In 2000 cases, 1835 (91.7%) met MDS criteria for PD, either clinically established (n = 1261, 63.1%) or clinically probable (n = 574, 28.7%), leaving 165 (8.3%) not fulfilling criteria. Clinically established cases were significantly more likely to have limb rest tremor (89.3%), a good l-dopa response (79.5%), and olfactory loss (71.1%), than clinically probable cases (60.6%, 44.4%, and 34.5% respectively), but differences between probable PD and ‘not PD’ cases were less evident. In cases not fulfilling criteria, the mean MDS UPDRS3 score (25.1, SD 13.2) was significantly higher than in probable PD (22.3, SD 12.7, p = 0.016) but not established PD (22.9, SD 12.0, p = 0.066). The l-dopa equivalent daily dose of 341 mg (SD 261) in non-PD cases was significantly higher than in probable PD (250 mg, SD 214, p < 0.001) and established PD (308 mg, SD 199, p = 0.025). After 30 months' follow-up, 89.5% of clinically established cases at baseline remained as PD (established/probable), and 86.9% of those categorized as clinically probable at baseline remained as PD (established/probable). Cases not fulfilling PD criteria had more severe parkinsonism, in particular relating to postural instability, gait problems, and cognitive impairment.

**Conclusion:**

Over 90% of cases clinically diagnosed as early PD fulfilled the MDS criteria for PD. Those not fulfilling criteria may have an atypical parkinsonian disorder or secondary parkinsonism that is not correctly identified by the UK Brain Bank criteria, but possibly by the new criteria.

## Highlights

• The MDS diagnostic criteria for Parkinson's Disease were tested retrospectively in a large cohort study.
• Over 90% of cases diagnosed clinically as PD fulfilled MDS diagnostic criteria for PD.
• Over 60% were categorized ‘Clinically established PD’; under 30% were ‘Clinically probable PD’.
• Cases categorized as ‘not PD’ (under 10%) had more severe, less therapy responsive parkinsonism.
• Categorization as PD by the MDS criteria was 85% stable after 2.5 years of follow-up.

## 1 Introduction

The accurate diagnosis of Parkinson's disease (PD) assists patient management and healthcare planning, and the identification of effective new treatments, which is important for a disease with an increasing prevalence [1]. Clinical diagnostic accuracy is suboptimal, being around 80% based on an overview of 11 studies [2] [3]. As there is no biomarker or specific imaging test for PD, the diagnosis relies heavily on clinical assessment [4]. Increased knowledge about PD and disorders that mimic it has allowed the development of new clinical Movement Disorder Society (MDS) diagnostic criteria [4]. These retain the core definition of parkinsonism (bradykinesia, rigidity and/or rest tremor) but do not allow for postural instability, compared to the United Kingdom (UK) Brain Bank criteria [5]. After confirmation of parkinsonism, a clinical diagnosis of PD according to the MDS criteria is based on: absolute exclusion criteria (which rule out PD), red flags (which must be counterbalanced by supportive criteria), and positive supportive criteria. These are combined to determine diagnostic certainty as clinically probable PD, or clinically established PD [4]. The new consensus criteria represent a summation of available knowledge, but have not been tested prospectively, which was the purpose of the current study. We classified and described the phenotype of cases recruited to an observational study of PD, according to fulfilment of the new MDS criteria [4].

## 2 Methods

Patients were recruited to *Tracking Parkinson's*, a large prospective, UK multicenter project, as detailed elsewhere [6]. In brief, recent onset PD cases with a clinical diagnosis and fulfilling UK Brain Bank criteria at study entry [5] were recruited, including drug-naïve and treated patients. Those with severe comorbid illness, other degenerative parkinsonism, symmetrical lower body parkinsonism, drug-induced parkinsonism, or a clinical diagnosis of dementia at first assessment were excluded. Levodopa (l-dopa) equivalent daily doses (LEDD) were calculated using an established formula [7]. Motor subtypes were determined by established methods [8]. Montreal cognitive assessment (MoCA) scores were adjusted for years of education and categorized as normal (>23), mild cognitive impairment (MCI) (22–23, or less than 22 but without functional impairment), or dementia (21 or less with functional impairment) [9]. Olfaction testing used either the 40-item University of Pennsylvania Smell Identification Test (UPSIT) or Sniffin’ Sticks 16-item version (SS), and hyposmia was defined as previously reported [10]. FP-CIT scanning was performed as part of routine care, on the basis of diagnostic uncertainty.

As the MDS diagnostic criteria were published after patient recruitment was complete, the criteria were applied retrospectively. Each component was mapped to the assessments performed, including MDS UPDRS, lying and standing blood pressure, response to l-dopa test dose, non-motor symptom scales, scales for outcome in autonomic symptoms in PD, PD and Epworth sleep score, and questionnaires for wearing off, rapid eye movement behavior disorder, constipation, Leeds anxiety and depression, and PD quality of life. Clinicians assessed each case, at baseline (study entry) and after 1 and 2.5 years, for any unusual or atypical features for PD, under several categories: clinical presentation, symptoms, signs, disease course, or therapy response. To ensure that early signs were not overlooked, such features were noted when they ‘could indicate an alternative diagnosis to PD (i.e. idiopathic parkinsonism with the presence of Lewy bodies in the substantia nigra), no matter how remote’. Clinicians also rated their clinical diagnostic certainty between 0% (not PD) and 100% (definite PD).

There was some variance in the data elements collected, compared to the MDS criteria: we recorded vertical gaze palsy (rather than only downward vertical gaze palsy), and did not specifically note recurrent falls, inspiratory stridor, or frequent inspiratory sighs. We assessed for the absence of an observable l-dopa response following MDS criteria (daily l-dopa dose 600 mg or more, and bradykinesia or rigidity in at least one body part exceeding 2 points), and carried out an additional exploratory analysis (no l-dopa dose threshold, MDS UPDRS 3 score above 20 to define at least moderate disease, and clinician assessment of ‘little or no response to l-dopa or a dopamine agonist’). For assessment of a clear and dramatic response to dopaminergic therapy, we used an improvement of over 30% in MDS UPDRS 3 after the patient's usual morning l-dopa dose, taken after a practically defined overnight period off medication.

### 2.1 Statistical analysis

Regression models were used to test the association between the three MDS classification groups and clinical features. Clinical characteristics were the dependent variables and the MDS criteria groups (along with age, gender and disease duration) were the independent variables. Regression was linear for continuous outcomes, logistic for binary outcomes, ordinal (also called proportional odds) for ordinal outcomes (MoCA and Hoehn and Yahr stage), and multinomial for motor subtype (using tremor dominant as the baseline category). Two-way p-values across the three MDS classification groups were calculated as 2-tailed, after adjustment for three confounders: age, gender and disease duration. The linearity of age and disease duration was tested using fractional polynomials in univariate models, and then transformed if non-linear. The results were not corrected for multiple comparisons. The agreement between baseline and follow-up categorization was tested using weighted kappa, and also, because of imbalance of group sizes and numbers of cases changing category, by the weighted Gwet AC1/AC2 coefficient [11], [12]. Statistical analysis was conducted using STATA (version 14, StataCorp, Texas, USA).

## 3 Results

There were 2000 cases at study entry, mean age 64.4 years (SD 9.8), disease duration 1.3 years (SD 0.9), and 64.9% were male. 1835 (91.7%) met the MDS diagnostic criteria for PD, either clinically established (n = 1261, 63.1% of all cases) or clinically probable (n = 574, 28.7% of all cases), leaving 165 (8.3% of all cases) who did not meet criteria (Table 1). Tremor as a symptom at onset was significantly more prevalent in clinically established PD (83.3%) than clinically probable PD (57.4%), or those not fulfilling criteria for PD (62.6%), both p < 0.001, and the proportion with a tremor dominant motor subtype followed the same pattern (Table 1). Cognition was worse in non-PD cases (21.7% dementia) compared to 15.5% dementia in clinically probable PD cases (p = 0.013) and 13.0% dementia in clinically established PD cases (p = 0.02). The MDS UPDRS 3 score was very similar for clinically established (22.9, SD 12.0) and clinically probable PD cases (22.3, SD 12.7), but was significantly higher in cases not fulfilling PD (25.1 SD 13.2, p = 0.016 compared to clinically probable PD). The LEDD in the cases failing to meet MDS criteria for PD was 341 mg (SD 261), which was significantly higher than those with clinically probable PD (250 mg, SD 214, p < 0.001), and in those with clinically established PD (308 mg, SD 199, p = 0.025).

**Table 1:** Demographic and disease features in 2000 cases with a clinical diagnosis of recent onset Parkinson's disease, categorized according to fulfilment of MDS diagnostic criteria for PD.

Characteristic	All cases N = 2000 (100%)	Fulfilment of MDS criteria for PD			Adjusted p-values		
		Not PD N = 165 (8.3%)	Clinically probable N = 574 (28.7%)	Clinically established N = 1261 (63.1%)	Not PD vs Clinically probable	Not PD vs Clinically established	Clinically probable vs Clinically established
Age at onset^a^	64.4 (9.8)	64.7 (9.7)	65.2 (9.9)	64.0 (9.7)	0.61^f^	0.39^f^	0.021^f^
Age at diagnosis^a^	66.2 (9.3)	66.5 (9.0)	67.1 (9.4)	65.8 (9.3)	0.49^f^	0.41^f^	0.010^f^
Age at baseline^a^	67.6 (9.3)	67.8 (9.0)	68.3 (9.4)	67.2 (9.3)	0.49^f^	0.41^f^	0.010^f^

Disease duration^a^	1.3 (0.9)	1.4 (0.9)	1.2 (0.9)	1.4 (0.9)	0.084^g^	0.77^g^	<0.00^g^

Gender (Male)^b^	1299 (64.9%)	104 (63.0%)	369 (64.3%)	826 (65.5%)	0.80^h^	0.51^h^	0.52^h^
Symptoms at onset							
Tremor^b^	1452 (74.4%)	102 (62.6%)	310 (57.4%)	1040 (83.3%)	0.19^i^	<0.001^i^	<0.001^i^
Rigidity^b^	1337 (71.9%)	114 (76.0%)	365 (70.1%)	858 (72.2%)	0.15^i^	0.32^i^	0.37^i^
Bradykinesia^b^	1493 (78.5%)	128 (81.5%)	411 (77.1%)	954 (78.7%)	0.21^i^	0.43^i^	0.35^i^
Postural instability^b^	364 (19.8%)	48 (31.0%)	114 (22.0%)	202 (17.4%)	0.012^i^	<0.001^i^	0.083^i^

Motor Subtype^c^							
Tremor Dominant	832 (45.9%)	46 (31.3%)	210 (41.3%)	576 (49.8%)			
PIGD	745 (41.1%)	86 (58.5%)	244 (47.9%)	415 (35.9%)	0.022^i^	<0.001^i^	<0.001^i^
Indeterminate	236 (13.0%)	15 (10.2%)	55 (10.8%)	166 (14.3%)	0.54^i^	0.69^i^	0.65^i^

MoCA^d^							
Normal	1342 (73.2%)	100 (65.8%)	386 (74.1%)	856 (73.7%)	0.013^i^	0.020^i^	0.59^i^
MCI	227 (12.4%)	19 (12.5%)	54 (10.4%)	154 (13.3%)			
Dementia	265 (14.4%)	33 (21.7%)	81 (15.5%)	151 (13.0%)			

MDS UPDRS 3^a^	22.9 (12.3)	25.1 (13.2)	22.3 (12.7)	22.9 (12.0)	0.016^i^	0.066^i^	0.22^i^

Hoehn and Yahr^e^							
0–1.5	948 (47.9%)	69 (41.8%)	283 (50.8%)	596 (47.4%)	0.063^i^	0.25^i^	0.18^i^
2–2.5	894 (45.2%)	69 (41.8%)	239 (42.9%)	586 (46.6%)	<0.001^i^	<0.001^i^	0.72^i^
3 +	137 (6.9%)	27 (16.4%)	35 (6.3%)	75 (6.0%)			

Untreated^b^	196 (9.8%)	17 (10.3%)	92 (16.1%)	87 (6.9%)	0.11^i^	0.12^i^	<0.001^i^

LEDD (mg per day)^a^	294 (211)	341 (261)	250 (214)	308 (199)	<0.001^i^	0.025^i^	<0.001^i^

Data are shown as mean and standard deviation or n%. MDS = Movement Disorder Society, PD = Parkinson's disease, UPDRS 3 = Unified Parkinson's disease rating scale Part 3, LEDD = levodopa equivalent daily dose, PIGD = postural instability and gait difficulty, MoCA = Montreal Cognitive Assessment.

a Linear regression.

b Logistic regression.

c Multinomial regression.

d Proportional odds regression.

e Partial proportional odds regression (failed proportional odds assumption for MDS criteria groups).

f Adjusted for sex and disease duration.

g Adjusted for age and sex.

h Adjusted for age and disease duration.

i Adjusted for sex, age and disease duration.

The numbers of red flags, supporting criteria, and absolute exclusions categorized by MDS diagnostic group are in Table 2. Most non-PD cases were categorized on the basis of one or more absolute exclusion (149 of 165 cases, 90.3%), rather than having an excess of red flags over supporting features (15 of 165, 9.1%), or having >2 red flags (1 of 165, 0.6%). In these non-PD cases, the most common exclusion criteria were vertical gaze palsy (n = 117, 70.9% of cases not meeting PD criteria, or 5.8% of all cases) and cerebellar features (n = 25, 15.2% of cases not meeting PD criteria, or 1.3% of all cases). Only 3 cases (0.2%) were excluded (and thereby categorized as non-PD) on the basis of an absent l-dopa response defined by the MDS criteria. However, using our alternative definition (at least moderate disease and subjectively absent or poor dopaminergic therapy response), 72 cases (3.6%) were categorized as non-PD, which increased the proportion of non-PD cases from 8.3% to 11.2%.

**Table 2:** Fulfilment of MDS criteria in 2000 cases with a clinical diagnosis of recent onset Parkinson's disease.

Characteristic	All cases N = 2000 (100%)	Fulfilment of MDS criteria for Parkinson's disease		
		Not PD N = 165 (8.3%)	Clinically probable N = 574 (28.7%)	Clinically established N = 1261 (63.1%)
Number of red flags				
0	1712 (85.6%)	111 (67.3%)	340 (59.2%)	1261 (100%)
1	251 (12.6%)	41 (24.8%)	210 (36.6%)	Not applicable
2	36 (1.8%)	12 (7.3%)	24 (4.2%)	Not applicable
>2	1 (0.1%)	1 (0.6%)	0 (0.0%)	Not applicable

Number of supporting criteria				
0	55 (2.8%)	13 (7.9%)	42 (7.3%)	Not applicable
1	387 (19.4%)	43 (26.1%)	344 (59.9%)	Not applicable
2	874 (43.7%)	65 (39.4%)	92 (16.0%)	717 (56.9%)
>2	684 (34.2%)	44 (26.7%)	96 (16.7%)	544 (43.1%)

Number of absolute exclusion criteria				
0	1851 (92.6%)	16 (9.7%)	574 (100.0%)	1261 (100%)
1	143 (7.2%)	143 (86.7%)	Not applicable	Not applicable
>1	6 (0.3%)	6 (3.6%)	Not applicable	Not applicable

Red flags				
Rapid gait progression	3 (0.2%)	0 (0.0%)	3 (0.5%)	Not applicable
Absence of motor progression	0 (0.0%)	0 (0.0%)	0 (0.0%)	Not applicable
Bulbar dysfunction	115 (5.8%)	29 (17.6%)	86 (15.0%)	Not applicable
Respiratory dysfunction	0 (0.0%)	0 (0.0%)	0 (0.0%)	Not applicable
Severe autonomic	148 (7.4%)	25 (15.2%)	123 (21.4%)	Not applicable
Recurrent falls	0 (0.0%)	0 (0.0%)	0 (0.0%)	Not applicable
Disproportionate anterocollis	0 (0.0%)	0 (0.0%)	0 (0.0%)	Not applicable
Common non-motor absent	0 (0.0%)	0 (0.0%)	0 (0.0%)	Not applicable
Pyramidal signs	36 (1.8%)	9 (5.5%)	27 (4.7%)	Not applicable
Symmetric parkinsonism	24 (1.2%)	5 (3.0%)	19 (3.3%)	Not applicable

Supporting criteria				
Clear l-dopa response	1363 (68.2%)	105 (63.6%)	255 (44.4%)	1003 (79.5%)
l-dopa induced dyskinesia	94 (4.7%)	7 (4.2%)	22 (3.8%)	65 (5.2%)
Rest tremor of a limb	1585 (79.3%)	111 (67.3%)	348 (60.6%)	1126 (89.3%)
Olfactory loss	1181 (59.0%)	87 (52.7%)	198 (34.5%)	896 (71.1%)

Absolute exclusion criteria				
Unequivocal cerebellar	25 (1.3%)	25 (15.2%)	Not applicable	Not applicable
Vertical gaze palsy	117 (5.8%)	117 (70.9%)	Not applicable	Not applicable
Fronto-temporal dementia/PPA	0 (0.0%)	0 (0.0%)	Not applicable	Not applicable
Parkinsonism in lower limbs only	1 (0.1%)	1 (0.6%)	Not applicable	Not applicable
Dopamine blocker/depleter	2 (0.1%)	2 (1.2%)	Not applicable	Not applicable
Absence of l-dopa response	3 (0.2%)	3 (1.8%)	Not applicable	Not applicable
Cortical sensory loss/apraxia	8 (0.4%)	8 (4.8%)	Not applicable	Not applicable
Normal functional dopamine imaging	0 (0.0%)	0 (0.0%)	Not applicable	Not applicable
Alternative parkinsonism documented	0 (0.0%)	0 (0.0%)	Not applicable	Not applicable

MDS = Movement Disorder Society, PD = Parkinson's disease, PPA = Primary progressive aphasia.

Considering the positive supportive MDS criteria, these were most prevalent in clinically established PD, and were considerably lower in clinically probable PD, but intermediate in those not meeting PD criteria (Table 2). Red flags were present in 288 of the 2000 cases (14.4%), of which the majority (234 cases, 81.3% of 288) were categorized as clinically probable PD (rather than non-PD) because of supportive features, reflecting the balancing approach in the MDS criteria [4] (Table 2). There were 2 positive supportive criteria in 56.9% of the 1261 clinically established PD cases, and more than 2 such criteria in 43.1% of these cases. These supportive criteria were less common in clinically probable PD (2 criteria in 16.0%, more than 2 criteria 16.7%), but were intermediate in those categorized as non-PD (2 criteria in 39.4%, more than 2 criteria in 26.7%).

After a mean follow-up of 2.5 (SD 0.6) years, the categorization of cases by MDS criteria as PD versus not PD was largely stable, compared to the baseline categorization (Table 3). Out of 165 non-PD at baseline, 156 (94.5%) remained as non-PD, and 9 (5.5%) were categorized as probable PD because of emergent supportive features, which balanced red flags. Clinically probable PD became clinically established PD due to the increased supporting features (to 2 or more) without any red flags (147 of 574 cases, 25.6%). Of the 1261 clinically established PD cases at baseline, 152 (12.1%) became clinically probable PD at follow-up, due to red flags emerging. Clinically probable PD cases at baseline remained probable, or became established, in 86.9% of cases. Clinically established PD cases at baseline remained established PD, or became clinically probable PD, in 89.5%. The number of cases categorized as not PD increased from 165 (8.3%) at baseline to 364 (18.2%) at follow-up. The overall percent agreement comparing categorization at baseline to follow-up was 74.2% (actual), or 83.8% (weighted). The weighted Kappa was 0.55, while the weighted Gwet AC1/AC2 was 0.70.

**Table 3:** Stability of MDS categorization of Parkinson's disease, comparing baseline and 2.5 years' follow-up.

Baseline analysis	Follow-up analysis			
	Not PD	Probable PD	Established PD	% probable or established PD
Not PD N = 165	156	9	0	5.5%
Probable PD N = 574	75	352	147	86.9%
Established PD N = 1261	133	152	976	89.5%

MDS = Movement Disorder Society, PD = Parkinson's disease.

During follow-up, 31 cases had a change in clinical diagnosis, of which 21 cases were: MSA (n = 6), PSP (n = 5), essential or dystonic tremor (n = 3), vascular parkinsonism (n = 2), and single cases of corticobasal degeneration, functional parkinsonism, multiple sclerosis, post-polio syndrome, and spinal cord compression (Suppl Table 1). The 10 remaining cases had inconclusive diagnoses: 4 with normal presynaptic dopaminergic functional imaging performed after study entry, and 6 not otherwise specified. Of the 31 cases with a revised diagnosis, 5 (16.1%) had been classified as non-PD by MDS criteria at baseline, which increased to 11 (35.5%) at follow-up; 12 (38.7%) were classified as clinically probable PD at baseline, which declined to 8 (25.8%) at follow-up; and 14 (45.2%) were classified as clinically established PD at baseline, which declined to 12 (38.7%) at follow-up.

The clinicians' assessment reported atypical clinical features that might raise diagnostic doubt in 181 cases (9.1%), and this was more common in cases categorized as non-PD by the MDS criteria (15.8%), compared to 12.0% in clinically probable cases, and 6.8% in clinically established cases. Clinicians rated the diagnostic certainty of PD at less than 90% in 521 cases (26.1%); 29.7% of the MDS non-PD cases had this <90% diagnostic certainty score, compared to 33.3% of those classified as clinically probable PD, and 22.3% of clinically established PD.

## 4 Discussion

Our study is the first to apply the new MDS diagnostic criteria for PD to a large scale cohort. We found that over 90% of patients, at an early disease stage and with cardinal motor features and a clinical diagnosis of PD, fulfilled the MDS criteria for PD at baseline (study entry), and a higher proportion was categorized as clinically established PD (more than 60%) than clinically probable PD (less than 30%). In our cohort, the MDS diagnostic criteria are therefore at least 90% sensitive compared to the most commonly used preceding criteria [5]. We found that categorization as not PD (under 10%) resulted almost exclusively from the presence of absolute exclusion criteria, rather than having more than 2 diagnostic red flags (only 1 case). Also, baseline categorization as ‘not PD’ affected one quarter of 31 cases with a later revised diagnosis, and this ‘not PD’ status increased to over 40% of these 31 cases at 2.5 years.

There were significant phenotypic differences between clinically established and clinically probable PD cases. Clinically established PD cases had more supporting diagnostic features than clinically probable PD cases, indicating that, when red flags are present, there are also fewer supporting criteria (by definition all clinically established PD cases have 2 or more supporting criteria; only around one third of clinically probable PD have 2 or more supporting criteria). Since rest tremor is one of the 4 supporting criteria, clinically established PD cases were therefore more likely to be tremor dominant, and less likely to have postural instability gait difficulty (PIGD) [8]. Clinically established PD cases were also more likely to have commenced anti-parkinsonian medication, and were prescribed higher doses of dopaminergic therapy (around 25% greater than clinically probable cases).

Given the critical significance of dopaminergic responsiveness to diagnostic accuracy [2], [3], both the supporting feature of a clear and dramatic response to dopaminergic therapy, and the absolute exclusion of an absent observable l-dopa response, are of particular importance. A good l-dopa response was present in those classified as clinically established PD (around 80%), but a significant proportion of cases (almost two-thirds) classified as non-PD also showed a good l-dopa response. This may reflect the known early-stage dopa-responsiveness in disorders such as PSP [13], [14] and MSA [15], which wanes over time. Absence of the l-dopa response by MDS criteria involved very few cases (0.2%), largely because the required daily l-dopa dose of at least 600 mg for this criterion was rare at this early disease stage. Our exploratory more inclusive definition of poor dopaminergic responsiveness identified more cases and increased the proportion of non-PD cases by around 3%. We will test whether this is a useful early indicator of a diagnosis other than PD during further follow-up.

We also found that disease severity was significantly greater for cases categorized as non-PD compared to PD cases, including worse motor severity, more cases with PIGD, and more cases with dementia. This replicates the baseline features in the 8.1% of 800 cases who entered the DATATOP study as PD but later underwent diagnostic revision to ‘not PD’, during 6 years mean follow-up [16]. In addition, the dopaminergic therapy dose was greater in ‘not PD’ cases using the MDS criteria. The cases identified by MDS criteria as atypical for PD therefore have more severe parkinsonism that is less responsive to dopaminergic therapy, suggesting that a majority of such cases have an atypical parkinsonian syndrome or comorbidity (e.g. cerebrovascular disease) [17], [18].

The proportion of cases with a revised diagnosis during follow-up (1.6%) was considerably lower than the number of cases categorized either at baseline (8.3%) or after follow-up (18.2%) as non-PD by MDS criteria. However, our clinicians much more frequently recognized atypical features (9.1% of cases at baseline), suggesting that diagnostic revision is delayed until atypical features are more definite. However, clinical trials of emerging treatments, targeted to either abnormal alpha-synuclein or tau protein accumulation, would benefit from earlier distinction of these disorders. Our findings suggest that distinguishing features are present even at this relatively early stage, which is consistent with one previous long-duration clinical and autopsy study, in which early diagnostic clues were followed by definitive features, which improved the clinical accuracy (which was higher for PSP than for MSA) [19].

The new MDS diagnostic criteria set targets for accurate case identification: 90% of clinically established cases should have Lewy body associated PD, and 80% of clinically probable cases [4]. As an indicator of this, we found the diagnostic PD categories to be stable: cases in both established and probable groups retained a PD categorization of around 85% after 2.5 years. However, within the PD groups, there was movement in both directions (around 1 in 10 clinically established cases became clinically probable, and around a quarter of clinically probable cases became clinically established). This helps to quantify the likelihood of emerging red flags, and the development of increased numbers of supporting features, both of which are central to the MDS criteria definitions. We also found support for a further aim of the MDS diagnostic criteria: that only 3% of cases categorized as non-PD would actually have PD [4]. We found that 7 cases (3.1%) changed category from not PD to clinically probable PD, because of the emergence of additional supporting features; the long-term validity of these observations will be tested in follow-up.

Rest tremor is one of the cardinal motor signs of parkinsonism [20], and one of 4 supporting features for PD, when present in a limb, in the MDS criteria [4]. However, rest tremor can be present in dystonia [21], essential tremor [22], PSP [23], MSA [24], functional disorders [25] and after stroke [26]. Rest tremor was not specific for PD (possible or probable) in one autopsy study [2]. In our cohort, limb rest tremor was common (around 70% of cases) in ‘not PD’ cases, against around 60% of clinically probable cases (although in clinically established PD it was almost 90%). Accordingly the MDS criteria help to emphasise the importance of other clinical features (red flags and exclusions) that are inconsistent with a diagnosis of PD.

There are certain limitations to our study design. As the MDS criteria were published after our study completed patient recruitment, their application was retrospective, and there were some variations in definitions. By recording vertical gaze palsy, rather than downgaze palsy only, the accuracy of these observations is less than optimal, and the number of cases categorized as non-PD may be increased. Our objective measure of l-dopa responsiveness was based on the patient's usual morning l-dopa dose, which differs from the response after a change in medication defined by the MDS criteria. In addition, we did not have data regarding recurrent falls, inspiratory stridor, or disproportionate anterocollis, or results of imaging cardiac sympathetic denervation (although this is rarely applied), so we may have slightly overestimated clinically probable PD cases. We do not have autopsy data, but enrolment to the Parkinson's UK Brain Bank is a component of our study, so this will become available in future.

In conclusion, the MDS criteria for PD are useful for corroborating the diagnosis of PD, amongst cases fulfilling the core definition of parkinsonism and with a clinical PD diagnosis, and helpful in categorizing levels of diagnostic certainty. Cases not fulfilling MDS diagnostic criteria for PD have more severe parkinsonism, in particular relating to postural instability, gait problems, and cognitive impairment.

## Author contributions

NM: Data collection, analysis, manuscript writing and editing.

ML: Data analysis, manuscript writing and editing.

NB, RAB, DJB, HRM: Data collection, study design, manuscript editing.

JH, NW, NW: Study design.

YBS: Study design, data analysis plan, manuscript editing.

KAG, DGG: Study design, data collection, analysis, manuscript writing and editing.

## Financial disclosures

N Malek, KA Grosset, MA Lawton, N Williams, Y Ben-Shlomo: No conflicts of interest.

N Bajaj has received payment for advisory board attendance from UCB, Teva Lundbeck, Britannia, GSK, Boehringer, honoraria from UCB Pharma, GE Healthcare, Lily Pharma, Medtronic and an unrestricted educational grant from GE Healthcare.

RA Barker has received grants from Parkinson's UK, NIHR, Cure Parkinson's Trust, Evelyn Trust, Rosetrees Trust, MRC and EU along with payment for advisory board attendance from Oxford Biomedica and LCT, and honoraria from Lilley, Wiley and Springer.

DJ Burn has received grants from NIHR, Wellcome Trust, GlaxoSmithKline Ltd, Parkinson's UK, and Michael J Fox Foundation. He has acted as consultant for GSK.

J Hardy has received honoraria from Eisai, and grant support from MRC/Wellcome, Parkinson's UK, and the Michael J Fox Foundation.

H R Morris has received grants from Medical Research Council UK, Wellcome Trust, Parkinson's UK, Ipsen Fund, Motor Neurone Disease Association, Welsh Assembly Government, PSP Association, CBD Solutions and Drake Foundation, and payment for advisory board attendance and lectures from Teva, AbbVie, Boehringer Ingelheim, and GSK.

DG Grosset has received payment for advisory board attendance from AbbVie, and honoraria from UCB Pharma, GE Healthcare, Civitas, and Acorda.
