# Supplementary material for: Utility of the new Movement Disorder Society clinical diagnostic criteria for Parkinson's disease applied retrospectively in a large cohort study of recent onset cases
Source: Parkinsonism Relat Disord. 2017 Jul;40:40–6. doi: 10.1016/j.parkreldis.2017.04.006 (PMC5570813; doi:10.1016/j.parkreldis.2017.04.006)
Supplement: Supplementary data [file mmc1.docx]

Supplementary Table 1. Diagnostic categorization and features in 31 cases who had a revised clinical diagnosis.

| Revised diagnosis | Case number | MDS baseline  categorization | Number (type) of exclusions | Number of red flags | Number of supporting criteria |
| --- | --- | --- | --- | --- | --- |
| Multiple system atrophy | 1 | Established | 0 | 0 | 2 (+response, tremor) |
|  | 2 | Probable | 0 | 2 (bulbar, autonomic) | 2 (+response, tremor) |
|  | 3 | Probable | 0 | 0 | 0 |
|  | 4 | Probable | 0 | 0 | 1 (tremor) |
|  | 5 | Probable | 0 | 1 (autonomic) | 2 (+response, tremor) |
|  | 6 | Probable | 0 | 0 | 1 (olfaction) |
| Progressive supranuclear palsy | 1 | Established | 0 | 0 | 3 (+response, tremor, olfaction) |
|  | 2 | Established | 0 | 0 | 2 (+response, olfaction) |
|  | 3 | Established | 0 | 0 | 2 (+response, tremor) |
|  | 4 | Not PD | 1 (gaze palsy) | 0 | 1 (+response) |
|  | 5 | Not PD | 1 (gaze palsy) | 0 | 1 (+response) |
| Essential or dystonic tremor | 1 | Established | 0 | 0 | 2 (+response, tremor) |
|  | 2 | Probable | 0 | 0 | 1 (tremor) |
|  | 3 | Not PD | 1 (gaze palsy) | 0 | 1 (tremor) |
| Normal functional dopamine imaging | 1 | Established | 0 | 0 | 2 (+response, tremor) |
|  | 2 | Probable | 0 | 0 | 1 (tremor) |
|  | 3 | Probable | 0 | 0 | 1 (olfaction) |
|  | 4 | Probable | 0 | 1 (autonomic) | 2 (+response, olfaction) |
| Vascular parkinsonism | 1 | Established | 0 | 0 | 2 (+response, tremor) |
|  | 2 | Established | 0 | 0 | 2 (+response, tremor) |
| Corticobasal degeneration | 1 | Not PD | 2 (cerebellar, cortical sensory loss) | 0 | 1 (response) |
| Multiple sclerosis | 1 | Established | 0 | 0 | 2 (+response, olfaction) |
| Post-polio syndrome | 1 | Established | 0 | 0 | 2 (+response, olfaction) |
| Spinal cord compression | 1 | Established | 0 | 0 | 3 (+response, tremor, olfaction) |
| Functional | 1 | Established | 0 | 0 | 2 (+response, tremor) |
| Unknown but not PD | 1 | Established | 0 | 0 | 2 (tremor, dyskinesia) |
|  | 2 | Established | 0 | 0 | 3 (+response, tremor, olfaction) |
|  | 3 | Probable | 0 | 1 (autonomic) | 3 (+response, tremor, olfaction) |
|  | 4 | Probable | 0 | 0 | 1 (+response) |
|  | 5 | Probable | 0 | 1 (autonomic) | 1 (+response) |
|  | 6 | Not PD | 1 (gaze palsy) | 0 | 1 (tremor) |

MDS = Movement Disorder Society, PD = Parkinson’s disease, +response = positive response to L-Dopa, tremor = rest tremor
